# Arctigenin from *Fructus arctii* Exhibits Antiaging Effects via Autophagy Induction, Antioxidative Stress, and Increase in Telomerase Activity in Yeast

**DOI:** 10.3390/antiox13060684

**Published:** 2024-06-02

**Authors:** Siqi Chen, Yajing Li, Enchan Wu, Qing Li, Lan Xiang, Jianhua Qi

**Affiliations:** 1 College of Chemistry and Materials Science, Sichuan Normal University, Chengdu 610068, China; 20221201040@stu.sicnu.edu.cn; 2 College of Pharmaceutical Sciences, Zhejiang University, Yu Hang Tang Road 866, Hangzhou 310058, China; 12019045@zju.edu.cn (Y.L.); 22019019@zju.edu.cn (E.W.)

**Keywords:** *Fructus arctii*, arctigenin, autophagy, antioxidative stress, telomerase activity, lifespan

## Abstract

Aging is often accompanied by irreversible decline in body function, which causes a large number of age-related diseases and brings a huge economic burden to society and families. Many traditional Chinese medicines have been known to extend lifespan, but it has still been a challenge to isolate a single active molecule from them and verify the mechanism of anti-aging action. Drugs that inhibit senescence-associated secretory phenotypes (SASPs) are called “senomorphics”. In this study, arctigenin (ATG), a senomorphic, was screened from the Chinese medicine *Fructus arctii* using K6001 yeast replicative lifespan. Autophagy, oxidative stress, and telomerase activity are key mechanisms related to aging. We found that ATG may act through multiple mechanisms to become an effective anti-aging molecule. In exploring the effect of ATG on autophagy, it was clearly observed that ATG significantly enhanced autophagy in yeast. We further verified that ATG can enhance autophagy by targeting protein phosphatase 2A (PP2A), leading to an increased lifespan. Meanwhile, we evaluated the antioxidant capacity of ATG and found that ATG increased the activities of the antioxidant enzymes, thereby reducing reactive oxygen species (ROS) and malondialdehyde (MDA) levels to improve the survival of yeast under oxidative stress. In addition, ATG was able to increase telomerase activity by enhancing the expression of *EST1*, *EST2*, and *EST3* genes in yeast. In conclusion, ATG exerts anti-aging effects through induction of autophagy, antioxidative stress, and enhancement of telomerase activity in yeast, which is recognized as a potential molecule with promising anti-aging effects, deserving in-depth research in the future.

## 1. Introduction

The global population is undergoing a rapid increase in the proportion of elderly individuals, with the percentage of people aged 65 years and older projected to escalate from 10% in 2022 to 16% in 2050 [1]. With the aging of the global population, the mandatory retirement age has increased in many countries, but there has been no corresponding rise in health lifespan of older people as expected [2]. On the contrary, age-related diseases such as diabetes, cardiovascular disease, cancer, Alzheimer’s disease (AD), kidney failure and osteoarthritis are becoming more prevalent, and the huge drug expenditure of the elderly will bring great economic pressure to their families and society [1]. At present, although there are numerous drugs that can alleviate the symptoms of the above-mentioned diseases, those drugs cannot cure the diseases in essence [3]. On the other hand, delaying aging may be an effective strategy to control and prevent age-related diseases. Currently, senotherapeutics, a new generation of anti-aging drugs, is under development. It is categorized into two groups, senomorphics and senolytics. The former prevents the occurrence of senescence by interfering with senescing-related signals, while senolytics can specifically kill senescent cells [4]. Therefore, we focus on identifying potential senotherapeutics with anti-aging effects in the hope of achieving healthy aging.

Aging is a complex and changeable process, which is usually accompanied by the accumulation of harmful substances and the occurrence of a variety of chronic diseases [5]. The timely removal of damaged substances in cells can minimize the harm to physiological functions. Autophagy is known for its ability to break down damaged proteins, dysfunctional mitochondria, and even invasive microorganisms within cells, which is a crucial regulator for maintaining cellular homeostasis [6]. It plays an important role in prolonging life, and it has been shown that increasing autophagy can delay aging [6,7]. So far, about 16–20 core genes have been identified with a function of mediating autophagy, and autophagy-related proteins encoded by these genes play a key role in the formation of autophagosomes [6]. Therefore, it is critical to establish a reliable method to monitor and quantify autophagy. In particular, Atg8, a core autophagy protein which is able to produce conjugates that localize to phagosomes and autophagosomes after autophagy activation, is a crucial parameter in assessment of the progression of autophagy [8]. In this study, we used a yeast strain expressing the GFP-Atg8 protein for the monitoring of autophagy when it occurs through monitoring free GFP released by the cleavage of the GFP-Atg8 protein [9]. Additionally, the *ATG2* and *ATG32* genes, which encode Atg2 and Atg32 proteins, respectively, also play an important role in autophagy. Atg2 is a lipid transfer protein involved in the formation of autophagosomes [10], while Atg32, a mitophagy-related protein, could assemble with Atg8 and Atg11 on the surface of mitochondria to form the initiator of mitophagy [11].

ROS induces autophagy in response to nutrient deficiency. It is a kind of toxic metabolites produced by aerobic respiration of cells, mainly including superoxide anions (O^2−^), hydrogen peroxide (H_2_O_2_), and hydroxyl radicals (HO·) [12]. At low levels, ROS is capable to conduct redox-dependent signals essential for cell proliferation, differentiation, and aging [13]. However, excess ROS can lead to oxidative stress, causing damages on biological macromolecules such as lipids, proteins, carbohydrates, and DNA, thereby reducing cell viability [12]. Under normal conditions, the level of ROS is controlled by breaking down it through the in vivo protective enzyme systems superoxide dismutase (SOD), catalase (CAT), and glutathione peroxidase (GPx) in the cell [12]. Nevertheless, the balance between ROS and antioxidant enzymes will be disrupted as cells age, causing oxidative stress, which is considered as an important underlying mechanism of aging [14].

In addition to autophagy and oxidative stress, telomeres and telomerase are also one of the key factors related to aging. Telomeres are special nucleotide sequences located at the end of chromosomes, whose length will gradually shorten with cell division, thereby inducing cell senescence [15]. Meanwhile, telomerase, a ribonucleoprotein enzyme, is able to elongate telomeres through reverse transcription, alleviating telomere loss from human to yeast [16]. In *Saccharomyces cerevisiae*, telomerase is primarily composed of two regulatory subunits, Est1 and Est3, a catalytic subunit, Est2, and RNA subunit TLC1 [17]. Among them, Est1 and Est3 play a key role in telomere replication in vivo, whereas Est2 and TLC1 are able to reconstitute telomerase activity in vitro [18].

*Saccharomyces cerevisiae*, with about 30% of the genome conserved on humans, was popularly used for studying aging and age-related diseases due to its advantages of economy, short lifespan, and simple operation compared with other aging models [19]. The aging process of yeast is mainly measured by its replicative and chronological lifespan. In detail, replicative lifespan is determined by the number of limited divisions of a single yeast cell, while chronological lifespan is defined as the length of time that a quiescent culture survives [20]. The traditional method of testing yeast replicative lifespan is hard and time-consuming. Therefore, a special yeast mutant strain, K6001, derived from W303, which is characterized by the fact that only mother cells can divide until death on yeast peptone dextrose (YPD) agar plate, to simplify the manipulations of replicative longevity, was chosen as an experimental model [21].

*Arctium lappa* L., a biannual herb with multiple pharmacological effects, is mainly distributed in Jilin, Zhejiang, Liaoning, and Heilongjiang provinces [22]. It has been used as a traditional Chinese medicine for hundreds of years. Its mature seed, *Fructus arctii*, exhibits the effects of evacuating fever, ventilating lung, clearing rash, and detoxing, making it an effective medicine to treat wind and heat cold, measles, rubella, carbuncles, and swollen sores [23]. The main components of *Fructus arctii* are lignans, flavonoids, phenolic acids, triterpenes, volatile oils, and other chemical components, which have multiple pharmacological effects such as antitumor, antioxidant, hypoglycemic, and anti-inflammatory [22]. Total lignans isolated from *Fructus arctii* have been shown to reduce blood glucose and body weight in mice with type 2 diabetes [24]. In addition, it can effectively improve cognitive dysfunction in AD model mice [25]. In this study, yeast models were used to screen a compound with anti-aging potential from the traditional Chinese medicine *Fructus arctii*. Furthermore, we found that this compound exhibited anti-aging effects via inducing autophagy and exhibiting antioxidative stress effects.

## 2. Materials and Methods

### 2.1. General, Culture Medium and Cells

Related reagents and consumables used in this paper are listed in the Supplementary Materials.

YPD medium, 2% D-(+)-glucose (Sigma-Aldrich Co., St. Louis, MO, USA), 2% hipolypeptone (Nihon Pharmaceutical Co., Ltd., Tokyo, Japan), and 1% yeast extract (Oxoid Ltd., Basingstoke, Hants, UK); YPD agar plate, add 2% ager (Sigma-Aldrich Co., St. Louis, MO, USA) to YPD medium; D-(+)-galactose liquid medium, 3% D-(+)-galactose (Sangon Biotech, Shanghai, China), 2% hipolypeptone, and 1% yeast extract; synthetic defined (SD) medium, 0.17% yeast nitrogen base without amino acids and ammonium sulphate (BIDI Medical Device Shanghai Co., Ltd., Shanghai, China), 0.5% ammonium sulphate (Xilong Chemical Co., Ltd., Guangdong, China), and 2% glucose; synthetic complete glucose (SC) medium, add 17 amino acids and nucleotide molecules (Shanghai Aladdin Biochemical Technology Co., Ltd., Shanghai, China) to SD medium, the specific ratios of amino acids and nucleotides are listed in the Supplementary Table S1.

The K6001 yeast strain was gifted by Professor Michael Breitenbach (University of Salzburg, Salzburg, Austria). The K6001 strains of Δ*sod1*, Δ*sod2*, Δ*cat*, Δ*gpx*, Δ*atg2*, and Δ*atg32*; BY4741; and YOM38 containing pR316-*GFP-ATG8* plasmid were obtained from Professor Akira Matsuura (Chiba University, Chiba, Japan). The S288C strain was purchased from Hangzhou Baosai Biotechnology Co., Ltd., Hangzhou, China. The genotypes of all of the above yeast strains were presented in the Supplementary Table S2. PC12 cells were cultured in DMEM (Cellmax, New Taipei City, Taiwan) supplemented with 7.5% fetal bovine serum (Cellmax) and 10% horse serum (Gibco, Grand Island, NY, USA) and 1% antibiotic–antimycotic solution (Solarbio, Beijing, China) in a humidified incubator at 37 °C and 5% CO_2_. NIH/3T3 (ATCC CRL-1658) cells were obtained from MeilunBio, tested negative for microbial contamination, and were routinely authenticated with STR assay. NIH/3T3 cells were cultured in DMEM (Cellmax) supplemented with 10% fetal bovine serum (Cellmax) and 1% antibiotic–antimycotic solution (Solarbio) in a humidified incubator at 37 °C and 5% CO_2_.

### 2.2. Isolation and Purification of ATG

*Fructus arctii* was purchased from Beijing Tongrentang Wuhu Pharmacy (Wuhu, Anhui, China). A voucher specimen (no. 20, 231, 005) was preserved in Zhejiang University, Institute of Materia Medica. Dried *Fructus arctii* (10 g) was crushed and extracted by sonication with 100 mL of analytical grade methanol for 30 min (repeated twice). Then, the supernatants were pooled and concentrated under vacuum to obtain 625.5 mg of crude methanol extract. The crude methanol extract was then eluted with hexane/ethyl acetate (100:0, 80:20, 50:50, 20:80, and 0:100) on a silica gel opening column to obtain a total of five fractions. Active fraction 4 (38.9 mg) obtained from hexane/ethyl acetate (80:20) was continued to be purified with CH_3_OH/H_2_O (40:60, 50:50, 60:40, 70:30, 80:20, and 100:0) using an ODS open column. Of the six fractions obtained above, fraction 4 (5.8 mg) obtained from CH_3_OH/H_2_O (60:40) is the active molecule. Its structure was identified by ^1^H NMR spectroscopy (Supplementary Figure S1) and compared with the literature [26]. ^1^H NMR (500 MHz, CDCl_3_): δ _H_ = 6.82 (1H, d, *J* = 8.0 Hz), 6.75 (1H, d, *J* = 8.1 Hz), 6.64 (1H, d, *J* = 1.9 Hz), 6.61 (1H, dd, *J* = 8.0, 2.0 Hz), 6.55 (1H, dd, *J* = 8.1, 2.0 Hz), 6.46 (1H, d, *J* = 1.9 Hz), 4.14 (1H, dd, *J* = 9.1, 7.2 Hz), 3.88 (1H, dd, *J* = 9.1, 7.3 Hz), 3.85 (3H, s), 3.82 (3H, s), 3.81 (3H, s), 2.92 (2H, m), 2.66–2.47 (4H, m). HRESI-TOF-MS (M + H)^+^ *m*/*z* 373.1649, which was calculated for C_21_H_24_O_6_ (M + H)^+^ 373,1646.

### 2.3. Replicative and Chronological Lifespan Assay

The replicative lifespan assay was performed according to a previous study [7]. In brief, K6001 yeast strain stored in a −30 °C refrigerator was transferred into a 15 mL centrifuge tube, washed three times with phosphate buffer (PBS), and inoculated into 5 mL galactose liquid medium. After shaking culture for 24 h (180 rpm, 28 °C), 1 mL of yeast suspension was taken from it and washed three times with PBS. Then, approximately 4000 yeast cells were counted by a hemocytometer and evenly spread on YPD agar plates, which supplemented with 10 µM resveratrol (RES) or at concentrations of 1, 3, 10, and 30 µM ATG, respectively. After standing culture at 28 °C for 48 h, 40 microcolonies per group were randomly selected under the microscope (Olympus Corporation, Tokyo, Japan) to count the number of daughter cells produced by one mother cell. The determination of the replicative lifespan of yeast mutant strains with K6001 background (Δ*sod1*, Δ*sod2*, Δ*gpx*, Δ*cat*, Δ*atg2*, and Δ*atg32*) were similar to that of K6001 yeast. RES, a polyphenol, has anti-aging effects on a wide range of organisms [27], so we used RES as a positive control to demonstrate the reliability of the experimental results, and ethanol was used to dissolve the samples and served as a negative control.

The chronological lifespan assay method was similar to the previous study [7]. Firstly, BY4741 yeast was inoculated in 5 mL YPD, and incubated for 24 h with shaking (180 rpm, 28 °C). On day 0, yeast cells with initial OD_600_ value of 0.01 were added to 50 mL SC medium and treated with 1 µM rapamycin (Rapa) or ATG at doses of 1, 3, and 10 µM, followed by shaking culture. On day 3, about 200 yeast cells were dispersed on YPD agar plates and cultured at 28 °C for 48 h to count the number of the colony forming units (CFUs) that had grown from each group. Thereafter, the above steps were repeated every 5 days until the yeast has a survival rate of less than 10% (The survival rate = CFUs/CFUs on day 3 × 100%). The number of colonies on day 3 was defined as 100%.

### 2.4. Senescence-Associated β-Galactosidase (SA-β-Gal) Assay

To determine SA-β-Gal activity [28], approximately 50,000 PC12 or NIH/3T3 cells were seeded in each well of a 24-well plate and cultured in 5% CO_2_ at 37 °C for 24 h. Then, the cells were treated with different tested samples. The positive and negative controls used were 0.5% DMSO and Rapa, respectively. After 24 h, PC12 and NIH/3T3 cells were treated with 7.5 µM and 0.3 µM etoposide (Eto) for 2 days, respectively. Then, SA-β-Gal activity was evaluated using senescence β-Galactosidase staining kit (Beyotime Biotechnology, Shanghai, China) following the manufacturer’s instructions. After completion of SA-β-Gal staining, cells were examined under a bright-field microscope (Olympus BX-63, Tokyo, Japan), followed by quantification using ImageJ software (Version 1.42q, National Institutes of Health, Rockville, MD, USA) to measure the percentage of SA-β-Gal^+^ cells. Each group was measured over 3 regions and more than 150 cells were counted in each region.

### 2.5. Cell Proliferation Assay

For 5-Ethynyl-2′-deoxyuridine (EdU) incorporation assays [28], BeyoClick™ EdU-488 cell proliferation kit (Beyotime Biotechnology Inc., Shanghai, China) was used, and the assay was performed according to the manufacturer’s instructions. NIH/3T3 cells were cultured in different doses of ATG (1, 3, 10, 30 µM) and 100 nM Rapa as a positive control for 24 h and then treated with 0.3 µM Eto for 2 days. Then, cultured cells were labeled with EdU (10 µM) for 2 h and fixed with 4% formaldehyde for 15 min. After permeabilization, cells were treated with click additive solution for 30 min, then cells were counterstain with Hoechst 33342 for 10 min. After washing, EdU-positive cells were imaged by Olympus IX63 fluorescence microscope (Olympus Corporation, Tokyo, Japan) and quantified as percentage of the total number of cells.

### 2.6. Cell Viability Assay

3-(4,5-Dimethylthiazol-2-yl)-2,5-diphenyltetrazolium bromide (MTT) assay was performed to measure the cell viability [28]. In general, 5000 NIH/3T3 cells were seeded into each well of a 96-well plate and cultured in 5% CO_2_ at 37 °C for 24 h. Then, cells were incubated with different concentrations of ATG and Rapa (as a positive control) for one day. The negative control used was 0.5% DMSO. With or without treatment of 0.3 µM Eto for 2 days, 100 µL of serum-free DMEM containing 500 µg/mL MTT was added and incubated for 4 h followed by removal of medium carefully and addition of 100 µL of DMSO. The plates were read with the absorbance at 570 nm by using a plate reader (BioTek Synergy H1, Agilent, Santa Clara, CA, USA).

### 2.7. Visualization of Autophagy

In brief, YOM38 yeast containing the pRS316-*GFP-ATG8* plasmid was inoculated into YPD and incubated for 24 h with shaking in the dark. Then, the medium was replaced with SD medium. After mixing, 20 mL SD medium containing 300 µM RES or 1, 3, or 10 µM ATG were added with yeast with an initial OD_600_ value of 0.1, and the cells were cultured in the dark with shaking. After 22 h, SD medium was removed, and then Hoechst 33342 at a final concentration of 1 µg/mL was added for staining for 8 min under dark condition. Then, the Hoechst 33342 was removed from the background by washing three times with PBS. Finally, the cells were resuspended in 30% glycerol solution and autophagy-induced yeast were observed and imaged using a confocal fluorescence microscope (Olympus BX-51, Tokyo, Japan).

To test the effect of ATG treatment on autophagy in K6001 yeast, the K6001 was inoculated in 5 mL of galactose liquid medium and cultured for 24 h (180 rpm, 28 °C). Then, the yeast with an initial OD_600_ value of 0.1 was added to 20 mL galactose liquid medium containing the doses of 10 µM ATG, 30 µM LB-100 or 10 µM ATG combination of 30 µM LB-100, respectively, and incubated by shaking for 22 h. Subsequently, an appropriate amount of yeast was taken from each group and the subsequent experimental operations were carried out in the dark environment. The green detection reagent in the autophagy detection kit (Enzo Life Sciences, New York, NY, USA) was diluted with PBS at a ratio of 4:1000, and 100 µL staining solution was added to each group to mix and incubated at 37 °C for 1 h. Thereafter, the staining solution was removed and Hoechst 33342 with a final concentration of 1 µg/mL was added for staining for 8 min. Finally, after removing Hoechst 33342 from the background by washing with PBS, yeast cells were resuspended in 30% glycerol buffer for observation and photographing using the confocal fluorescence microscope.

### 2.8. Western Blot Analysis

Western blot analysis was performed on the basis of a previous report [7]. Briefly, each group of about 20–40 µg protein of samples was separated by electrophoresis on sodium dodecyl sulfate polyacrylamide gels and transferred to a polyvinylidene difluoride (PVDF) (Bio-Rad Laboratories Inc., Hercules, CA, USA) membrane. Subsequently, immunoblotted proteins were detected using specific antibodies: anti-GFP (#598, 1:1000, Medical & Biological Laboratories, Nagoya, Japan), p16 (#db12968, 1:1000, Hangzhou Daige Biotechnology Co., Ltd., Zhejiang, China), p21 (#AP021, 1:200, Shanghai Biyantian Biotechnology Co., Ltd., Shanghai, China), p53 (#db14679, 1:1000, Hangzhou Daige Biotechnology Co., Ltd., Zhejiang, China), or anti-β-actin (#CW0096, 1:1500, Beijing ComWin Biotech Co., Ltd., Beijing, China). And the secondary antibodies of horseradish peroxidase-linked goat anti-rabbit IgGs (#CW0103, 1:5000, Beijing ComWin Biotech Co., Ltd., Beijing, China) for GFP or goat anti-mouse IgGs (#CW0102, 1:5000, Beijing ComWin Biotech Co., Ltd., Beijing, China) for β-actin. Finally, the protein bands were subsequently developed using the ECL Western blot chemiluminescence kit (Beijing ComWin Biotech Co., Ltd., Beijing, China), and the blot density was quantified utilizing Image Lab software (Version 6.1, Bio-Rad, Hercules, CA, USA).

### 2.9. Drug Affinity Responsive Target Stability (DARTS) Assay

The procedure of DARTS was performed as previously reported [29]. The NIH/3T3 cells were collected and lysed in RAPI buffer (Beijing ComWin Biotech Co., Ltd., Beijing, China) containing 1% protease inhibitor (Beijing ComWin Biotech Co., Ltd., Beijing, China) and 1% phosphatase inhibitor (Abcam Cambridge Biomedical Campus, Cambridge, UK) for 15 min. The sample was centrifuged at 4 °C (12,000× *g*, 20 min) and the supernatant was collected as protein sample. The protein concentration was determined using the BCA kit and diluted to 2 µg/µL. The cell lysis supernatant was further incubated with DMSO or ATG at doses of 1, 10, 30, or 100 µM for 1 h at room temperature. The incubated cell lysates were then hydrolyzed with pronase E (MedChemExpress, Shanghai, China) diluted 1:100 in 1× TNC buffer (50 mM Tris-HCl pH 8.0, 50 mM NaCl, 10 mM CaCl_2_) for 20 min at room temperature in the dark. After incubation, a 5× SDS-PAGE loading buffer was added to each sample and heated at 100 °C for 8 min for Western blot. Immunoblotted proteins were detected using specific antibodies: anti-PP2A (#ab168350, 1:5000, Abcam Cambridge Biomedical Campus, Cambridge, UK) and anti-GAPDH (#CW0100M, 1:1000, Beijing ComWin Biotech Co., Ltd., Beijing, China).

### 2.10. Cellular Thermal Shift Assay (CETSA)

The CETSA procedure was performed as previously reported [30]. The NIH/3T3 cells were collected and cultured with ATG at a dose of 100 µM or DMSO for 1 h, washed three times with PBS, and collected in 15 mL centrifuge tubes. Then, cell suspensions were made by adding 1 mL of PBS containing 1% protease inhibitor to each sample. The cell suspensions were divided into 8 aliquots and heated at the corresponding temperature for 3 min, respectively. The heat-treated cell suspensions were repeatedly freeze-thawed three times with liquid nitrogen, centrifuged at 12,000× *g* for 20 min at 4 °C, and the supernatants were collected for Western blot. For isothermal dose-response CETSA experiment, the NIH/3T3 cell lysis supernatant was incubated with DMSO or ATG at doses of 1, 10, 30 or 100 µM for 2 h at room temperature and heated at 58 °C for 3 min. The supernatant of each sample was then collected by centrifugation at 4 °C (12,000× *g* for 20 min). Then, a 5 × SDS-PAGE loading buffer was added and heated at 100 °C for 8 min for Western Blot. Immunoblotted proteins were detected using specific antibodies: anti-PP2A and anti-β-actin.

### 2.11. Antioxidative Stress Test

BY4741 yeast cells with an initial OD_600_ value of 0.1 were added to 20 mL YPD with 0, 1, 3, and 10 µM ATG or 10 µM RES, and incubated at 28 °C for 24 h at 180 rpm with shaking. Afterwards, each group of yeast was diluted 10-fold with PBS, and 5 µL of each yeast suspension was dropped onto YPD agar plates containing 9.5 mM H_2_O_2_. The concentration of H_2_O_2_ was the optimal one that had been selected through pre-experiments. The growth of each group of yeast colonies was observed daily and photographed for recording after 3 days of incubation. To digitize the yeast antioxidant stress result, approximately 200 yeast cells treated with 10 µM RES or at concentrations of 0, 1, 3, and 10 µM ATG were coated on YPD agar plates with or without 6.0 mM H_2_O_2_. After 48 h of culture, the number of colonies grown in each group was counted separately. The survival rate of yeast cells was equal to the ratio of the number of colonies growing on H_2_O_2_ containing free mediums.

### 2.12. Determination of ROS and MDA Levels

The ROS level was measured as previously reported [7]. BY4741 with an initial OD_600_ value of 0.1 was treated with 10 µM RES or 1, 3, and 10 µM ATG for 24 h, respectively. After washing the yeasts three times with PBS, the fluorescent probe 2′,7′-dichlorodihydrofluorescein diacetate (DCFH-DA) was added with a final concentration of 10 µM and incubated for 1 h in the dark by shaking (180 rpm, 28 °C). Next, the yeast cells were harvested by centrifugation and washed three times with PBS for the purpose of removing DCFH-DA. The 2′,7′-dichlorodihydrofluorescein (DCF) fluorescence intensity of approximately 1 × 10^7^ yeast cells in each group at excitation wavelength of 488 nm and emission wavelength of 525 nm were determined using a Varioskan flash spectral scanning multimode reader (Thermo Fisher Scientific, Waltham, MA, USA).

In the measurement of MDA level, yeasts were cultured as ROS assay. After the yeast cells were washed three times with PBS, 500 µL of PBS and grinding beads were added and ground for 1 min at 70 Hz using an automated sample rapid grinder (Shanghai Jingxin Inc., Shanghai, China). Subsequently, the fragmented cells were centrifuged at 12,000× *g* for 10 min at 4 °C, and the supernatants were collected as protein samples. Protein concentrations were determined using a BCA kit (CoWin Biotech, Beijing, China). The content of MDA in yeast cells was determined according to the instructions of MDA assay kit (Nanjing Jiancheng Bioengineering Institute, Nanjing, China).

### 2.13. Determination of SOD, GPx, and CAT Antioxidant Enzyme Activities

For SOD, GPx, and CAT antioxidant enzyme activity assays, firstly, the BY4741 yeast strain was inoculated in YPD and incubated at 180 rpm and 28 °C for 24 h. Immediately afterwards, 10 µM RES or 1, 3, and 10 µM ATG were added to treat BY4741 yeast cells with an initial OD_600_ value of 0.1 and cultured at 28 °C with shaking for 24 h. Subsequently, cells from each group were collected separately, 500 µL PBS and grinding beads were added, and then they were sonicated for 1 min on ice. Proteins were collected by centrifugation at 12,000× *g* for 10 min at 4 °C. After determination of protein concentration, the protein concentration of each sample was diluted to 1.25 µg/µL for antioxidant enzyme activity determination. Finally, the enzyme activity in each group of yeast cells was determined according to the SOD (Nanjing Jiancheng Bioengineering Institute, Nanjing, China), GPx and CAT (Beyotime Biotech, Shanghai, China) antioxidant enzyme activity assay kit instructions. Detailed assay procedures are presented in the Supplementary Methods.

### 2.14. Telomerase Content Test

The S288C yeast strain was inoculated in 5 mL of YPD and incubated at 28 °C 180 rpm for 24 h. Subsequently, S288C yeast cells with an initial OD_600_ value of 0.1 were treated for 48 h with 10 µM RES or at doses of 1, 3, and 10 µM ATG. The extraction of yeast proteins as well as the determination of protein concentration were performed as mentioned above for the determination of SOD antioxidant enzyme activity. Finally, the protein concentration of each group was diluted to 10 µg/µL, and telomerase activity was measured according to the instructions for use of the Telomerase ELISA kit (Shanghai Tongwei Biotechnology Co., Ltd., Shanghai, China).

### 2.15. Real-Time Fluorescent Quantitative PCR (qRT-PCR)

The qRT-PCR was performed as previously described [7]. Briefly, S288C yeast cells with an initial OD_600_ value of 0.1 were treated for 24 h or 48 h by 10 µM RES or doses of 1, 3, or 10 µM ATG. Subsequently, the cells of each group were collected separately, and the total RNA was extracted by adding grinding beads and 1 mL of TRIzon Reagent (CoWin Biotech, Beijing, China) and grinding at 68 Hz for 3 min. Total RNA concentration was determined using a NanoDrop one Ultra-Micro Spectrophotometer (Thermo Scientific, Wilmington, DE, USA). The cDNA was subsequently synthesized by reverse transcription using 5 µg of total RNA and a HiFi-MMLV cDNA Kit (CoWin Biotech, Beijing, China). Primers design for qRT-PCR were performed using Primer Premier 6.0 software (Premier Inc., Kitchener, ON, Canada). The primers used were as follows:

Est1-F = 5′-TTCCGTGATACCATTGGTTCTG-3′, R = 5′-CGTCAGTGGATTACTCGTGTT-3′;

Est2-F = 5′-GGCTCAACGATCATCCTCATC-3′, R = 5′-ATGCGACAAGTCCAATACGG-3′;

Est3-F = 5′-TTGAAGACAACTCGGAGCAT-3′, R = 5′-ACTAAGTCAGCAT CGCCAATG-3′;

Tub1-F = 5′-CCAAGGGCTATTTACGTGGA-3′, R = 5′-GGTGTAATGGCCTCTTGCAT-3′.

qRT-PCR was performed using CFX96 Touch (BioRad, Hercules, CA, USA) with SYBR Premix EX Taq TM (Takara, Otsu, Japan). The thermal recycling parameters for yeast were as follows, *EST1*, *EST2* and *EST3*, 95 °C for 2 min, followed by 40 cycles, 95 °C for 15 s, 55 °C for 20 s, and 70 °C for 20 s. All results were standardized to *TUB1* level, and relative mRNA transcript levels were analyzed using the 2^−ΔΔCt^ formula.

### 2.16. Biostatistical Analysis

GraphPad Prism 8.0.2 software (GraphPad Software, San Diego, CA, USA) was used to analyze the experimental data. One-way ANOVA followed by Tukey’s multiple comparison test was used to analyze the significance differences between the groups. The log-rank (Mantel–Cox) test was used to analyze the chronological lifespan data of yeast. Each experiment was repeated three times, and data for each experiment are shown as mean ± SEM. 

## 3. Results

### 3.1. ATG Prolongs the Lifespan of Yeast and Relieves Eto-Induced Senescence of Mammalian Cells

**Figure 1:**
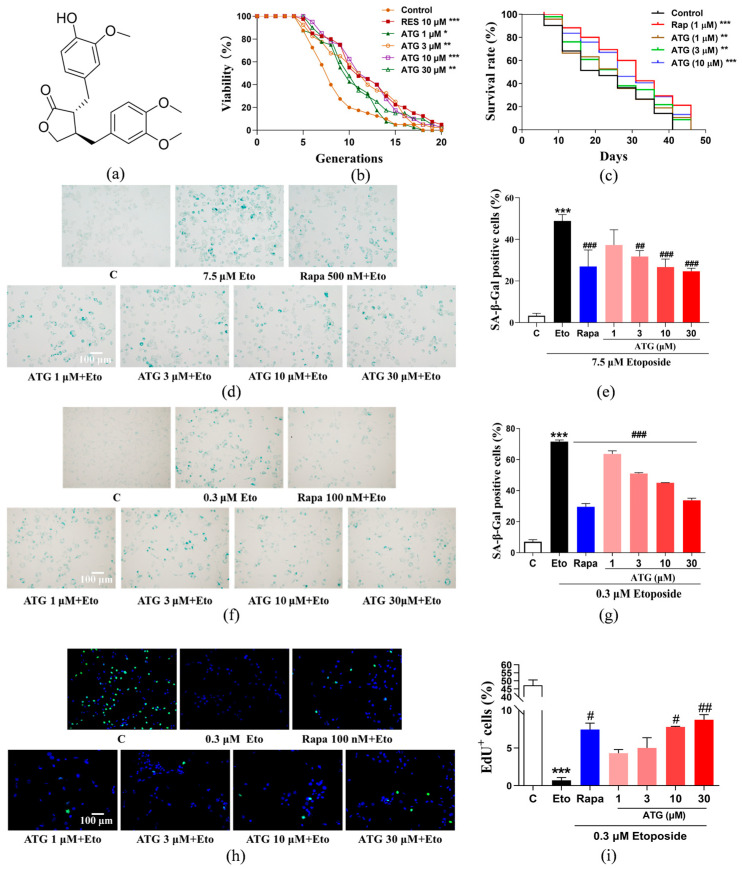
Chemical structure and anti-aging activity of ATG. (**a**) Chemical structure of ATG. (**b**) Effects of ATG at concentrations of 1, 3, 10, and 30 µM or RES at 10 µM on the replicative lifespan of K6001 yeast and (**c**) on the chronological lifespan of BY4741 yeast. (**d**) Anti-aging effect of ATG in Eto-induced senescence in PC12 cells. (**e**) The digital result of (**d**). (**f**) Anti-aging effect of ATG in Eto-induced senescence in NIH/3T3 cells. (**g**) The digital result of (**f**). (**h**) EdU staining of Eto-induced NIH/3T3 cells after ATG treatment. (**i**) The digital result of (**h**). #, ## and ### represent significant differences at *p* < 0.05, *p* < 0.01 and *p* < 0.001 compared with Eto group. *, **, and *** represent significant differences compared with the negative control group for *p* < 0.05, *p* < 0.01, and *p* < 0.001, respectively.

Yeast is a very crucial model in the study of aging and its mechanisms. In the current study, we used the replicative lifespan of a specific yeast strain K6001 to preliminarily test the anti-aging effect of ATG; its chemical structure is shown in Figure 1a. First, we tested the anti-aging activity of 10 µM RES or ATG at concentrations of 0.3, 1, 3, 10, 30, and 100 µM (Figure 1b and Supplementary Figure S2). The average lifespan of the negative control group was 7.75 ± 0.54; the positive group was 11.20 ± 0.71 (*p* < 0.001); and the mean lifespan of the 1, 3, 10, and 30 µM ATG treatment groups were 9.30 ± 0.53 (*p* < 0.05), 10.53 ± 0.69 (*p* < 0.01), 11.10 ± 0.61 (*p* < 0.001), and 10.25 ± 0.63 (*p* < 0.01), respectively. Thus, this molecule is a compound with longevity potential and deserves further study. Based on the results of yeast replicative lifespan, we found that the optimal concentration of ATG in yeast system was 10 µM, so in the subsequent yeast-related experiments, we usually selected 1, 3, and 10 µM as the doses of administration. Meanwhile, in the cellular model, we increased the dosing range appropriately, and 1, 3, 10, and 30 µM were selected as the administered doses. In addition, the anti-aging effect of ATG was investigated using the BY4741 yeast strain chronological lifespan. Rapa was used as a positive control due to its excellent performance in prolonging the chronological lifespan of yeast [31]. Treatment with different doses of ATG were able to significantly prolong yeast lifespan compared with the negative control (Figure 1c, *p* < 0.01, *p* < 0.01, and *p* < 0.001).

To further verify the anti-aging effects of ATG, we adopted two different mammalian cell lines, PC12 cell lines and NIH 3T3 cell line, by using Eto, a chemotherapy drug that can inhibit topoisomerase, destroy the reconnection of DNA supercoil dissociation chain, cause DNA damage, and lead to cell aging [32]. After that, we detected the changes on the biomarkers of senescent cells, such as SA-β-Gal, which is a β-galactosidase expressed in senescent cells and can be detected at pH 6.0; SA-β-Gal activity is now a widely used biomarker in studies of cellular senescence in culture and in vivo [33]. Two days after Eto administration, around half of the cells were SA-β-Gal positive (Figure 1d–g, *p* < 0.001, and *p* < 0.001). ATG in the dose range of 1 µM–30 µM and Rapa significantly reduced SA-β-Gal positive cells in PC12 cells (Figure 1d,e, *p* < 0.001, *p* < 0.01, *p* < 0.001, and *p* < 0.001) and NIH/3T3 cells (Figure 1f,g, *p* < 0.001, *p* < 0.001, *p* < 0.001, and *p* < 0.001), respectively.

Since stressed cells also showed positivity in the SA-β-Gal activity assay, and stressed cells may either recover from stress state or undergo senescence, assaying SA-β-Gal activity alone to demonstrate the anti-aging activity of ATG has limitations [33]. It is well known that cells gradually lose their ability to proliferate after entering senescence state, so we performed a cell proliferation assay using EdU to assess whether the cells had reached the senescent state after Eto induction [28]. As shown in the results, almost half of NIH/3T3 cells were shown EdU positive with green fluorescence in control group, while the percentage decreased to no more than 1% in Eto-treated cells (Figure 1h,i, *p* < 0.001). This means that cells induced by Eto almost completely lose their ability to proliferate. However, pre-treatment of ATG (10, 30 µM) and Rapa (100 nM) can retore the proliferative ability of cells to a certain extent.

### 3.2. ATG Acts as A Senomorphic

**Figure 2:**
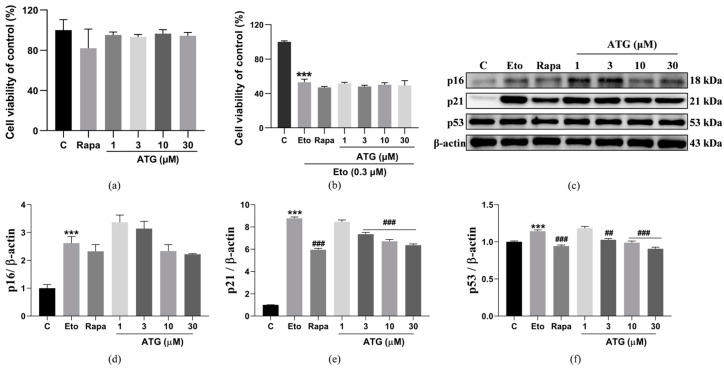
Antiaging therapeutic effects of ATG. (**a**) Toxicity of ATG in the dose range of 1–30 µM and Rapa at a dose of 100 nM to normal cells. (**b**) Toxicity of ATG in the dose range of 1–30 µM and Rapa at a dose of 100 nM to senescent cells. (**c**) Western blot of p16, p21, and p53 in Eto induced NIH/3T3 cells after ATG treatment. (**d**) The digital results of p16 in (**c**). (**e**) The digital results of p21 in (**c**). (**f**) The digital results of p53 in (**c**). ## and ### represent significant differences at *p* < 0.01 and *p* < 0.001 compared with Eto group. *** represent significant differences compared with the negative control group for *p* < 0.001.

To distinguish whether ATG belongs to which category of senotherapeutics, senolytics or senomorphics. We first examined the effect of ATG on NIH/3T3 cells viability using MTT assay. As shown in Figure 2a,b, ATG had no significant effect on cell viability in the range of 1–30 µM compared to control. For senescent cells induced by 0.3 µM Eto for 2d, ATG also had no significant effect on the viability of senescent cells compared with the Eto group. It has been reported that Rapa acts as senomorphics rather than senolytics [34], which is also consistent with our results. The results indicate that ATG did not specifically induce apoptosis in senescent cells, suggesting that it may be a molecule belonging to senomorphics. At the same time, we used Western blot to analyze the effect of ATG on the expression levels of p16, p21 and p53, which are related to cell senescence or cell cycle arrest in cells [35]. In ATG-treated Eto-induced NIH/3T3 cells (Figure 2c–f and Supplementary Figure S3), we observed a decrease in p21 (*p* < 0.001, *p* < 0.001, and *p* < 0.001) and p53 (*p* < 0.01, *p* < 0.001, and *p* < 0.001), but no significant difference in p16 expression level.

### 3.3. ATG Enhances Autophagy in Yeast

**Figure 3:**
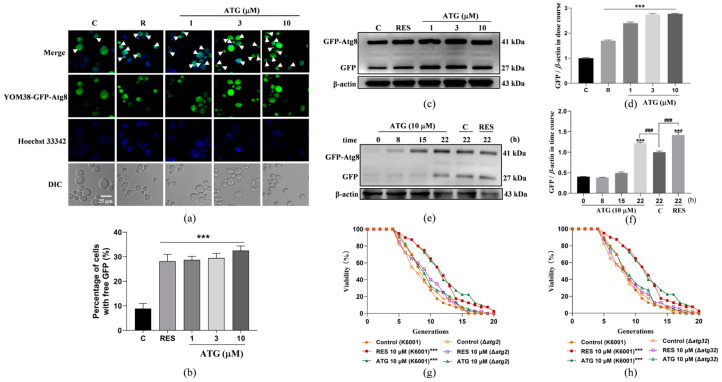
Effect of ATG on autophagy in yeast. (**a**) Fluorescence images of autophagy in YOM38 yeast cells after treatment with 300 µM RES or doses of 1, 3, and 10 µM ATG for 22 h, white arrowheads point to yeast cells undergoing autophagy. (**b**) Digital results of free GFP content in YOM38 yeast with 10 pictures with a cell number not less than 50; each group were randomly selected for statistical analysis. (**c**) Western blot analysis of GFP-Atg8 and free GFP in yeast cells after treatment with 300 µM of RES or different doses of ATG for 22 h in SD medium. (**d**) The digital results of (**c**). (**e**) Western blot analysis of GFP-Atg8 and free GFP in yeast cells treated with 300 µM RES or 10 µM ATG for specific periods of time. (**f**) The digital results of (**e**). (**g**,**h**) Replicative longevity results of the K6001 strain Δ*atg2* (**g**) and Δ*atg32* (**h**) treated with 10 µM RES or 10 µM ATG. The average lifespan in K6001 were 8.18 ± 0.47 in negative control, 10.98 ± 0.62 in positive treatment group (*p* < 0.001), and 11.23 ± 0.68 in the group treated with 10 µM ATG (*p* < 0.001). The average lifespan of Δ*atg2* mutant were 7.98 ± 0.54 in the negative group, 8.80 ± 0.57 in the positive group, and 8.83 ± 0.55 in the 10 µM ATG treatment group. The average lifespan of Δ*atg32* mutant were 7.92 ± 0.57 in the negative group, 8.38 ± 0.60 in the positive group, and 8.57 ± 0.55 in the 10 µM ATG treatment group. ### represent significant differences at *p* < 0.001 compared with control group in (**f**). *** represent significant differences compared with the negative control group for *p* < 0.001.

Autophagy is a key protective mechanism in eukaryotes that is able to degrade abnormally aggregated proteins and dysfunctional organelles, recycle useful substances, and benefit the health of the organism [6]. Therefore, we tested the effect of ATG on autophagy in yeast. We used the YOM38-*GFP-ATG8* yeast strain to evaluate the effect of different doses of ATG on yeast autophagy. Figure 3a is a fluorescence image of autophagy in yeast, and Figure 3b is the result after its digitization. It was clearly observed that the amount of free GFP in yeast was significantly increased after ATG treatment at the concentrations of 1, 3, and 10 µM. In parallel, we used Western blot technology to detect the free GFP flux released by yeast autophagy. After treatment with ATG for 22 h, the expression of free GFP protein was significantly increased. Among them, the content of free GFP protein in yeast increased most significantly after treatment with 10 µM ATG, so we selected it for yeast treatment to study the relationship between autophagy and time (Figure 3c,d and Supplementary Figure S4a). In the time-course of autophagy experiments, free GFP protein expression was time-dependent. Treatment with 10 µM ATG for 8 and 15 h showed almost no increase in autophagy, but a significant augment in free GFP protein expression was observed after 22 h of treatment (Figure 3e,f and Supplementary Figure S4b). *ATG2* and *ATG32* are genes associated with autophagy. For the purpose of exploring whether autophagy is responsible for prolonging the lifespan of yeast, we tested the effect of ATG on the replicative lifespan of Δ*atg2* and Δ*atg32* yeast strains with a K6001 background. As shown in Figure 3g,h, ATG failed to prolong the average lifespan of the yeast mutants described above, indicating that autophagy is involved in the anti-aging effect of ATG. In other words, ATG can prolong yeast lifespan by inducing autophagy.

### 3.4. LB-100, a PP2A Inhibitor Abolished the Life-Prolonging Effect of ATG in Yeast

**Figure 4:**
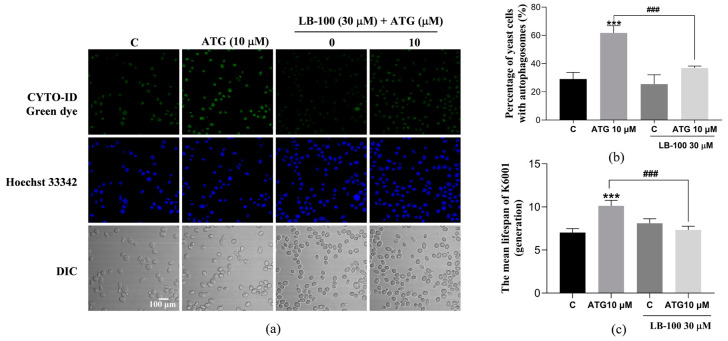
Effects of ATG and LB-100 on autophagy in K6001 yeast. (**a**) Effects of LB-100 on autophagy of K6001 yeast with ATG treatment. (**b**)The digital result of (**a**). (**c**) Effects of LB-100 on the replicative lifespan of K6001 yeast with ATG treatment. ### represent significant differences at *p* < 0.001 compared with ATG group in (**b**,**c**). *** represent significant differences compared with the negative control group for *p* < 0.001.

PP2A is a serine/threonine phosphatase involved in the regulation of multiple cellular processes [29]. It has been shown that it is essential for inducing autophagy after TORC1 inactivation, and ATG can enhance PP2A activity [29,36]. To explore whether the anti-aging effect of ATG is related to its enhancement of PP2A activity to induce autophagy, we used LB-100, a PP2A inhibitor, to verify it. Compared with the negative control group, treatment with ATG at a dose of 10 µM significantly increased the autophagy in K6001 yeast, and the effect of ATG was inhibited by LB-100 at a concentration of 30 µM (Figure 4a,b, *p* < 0.001 and *p* < 0.001). In addition, we tested whether the addition of LB-100 would eliminate the life-prolonging effect of ATG on K6001 yeast. As shown in Figure 4c, the average lifespan of K6001 yeast increased from 7.03 ± 0.46 to 10.13 ± 0.66 after treatment with 10 µM ATG (*p* < 0.001). The addition of 30 µM LB-100 alone had no effect on the average lifespan of yeast, but it was able to eliminate the anti-aging effect of ATG and reduced the mean lifespan of yeast from 10.13 ± 0.66 to 7.33 ± 0.51 (*p* < 0.001). These results suggest that ATG may enhance autophagy in yeast by increasing PP2A activity to achieve lifespan extension.

### 3.5. PP2A Is the Target Protein of ATG in NIH/3T3 Cells

**Figure 5:**
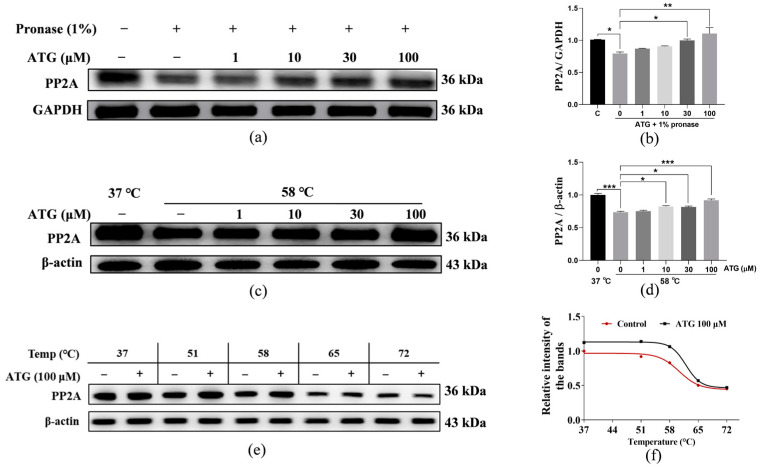
PP2A is a target protein of ATG. (**a**) NIH/3T3 cell lysates and various doses of ATG were incubated for 1 h at room temperature, digested with pronase E (0 or 1:100 dilution) for 20 min, and PP2A expression in the lysates was determined by Western blot. (**b**) The digital results of (**a**). (**c**)The changes in the protein PP2A in NIH/3T3 cells after administering of various concentrations of ATG and heating the samples at 58 °C. (**d**) The digital results of (**c**). (**e**) The changes in the protein PP2A in NIH/3T3 cells after treating with ATG at a dose of 100 µM and heating the samples at different temperatures, respectively. (**f**) The digital results of (**e**). *, **, and *** represent significant differences compared with the second group for *p* < 0.05, *p* < 0.01, and *p* < 0.001, respectively.

Identification of the target proteins of small molecule drugs is crucial to explore their mechanism of action. To identify potential target proteins of ATG, DARTS and CETSA techniques were used to test whether PP2A is the target of ATG. The DARTS technology is based on the fact that the binding of small molecules to target proteins increases the stability of proteins, thereby reducing proteins proteolysis [29], while CETSA technology is based on the principle that the binding of compounds to proteins affects the thermal stability of proteins [30]. The results of DARTS/Western bolt assay (Figure 5a,b and Supplementary Figure S5a, *p* < 0.05, *p* < 0.05,and *p* < 0.01) indicated that the stability of protein PP2A was improved by the addition of ATG. In addition, the CETSA/Western bolt results (Figure 5c–f, and Supplementary Figure S5b,c, *p* < 0.001, *p* < 0.05, *p* < 0.05,and *p* < 0.001) also showed that PP2A protein levels were significantly increased in the ATG-treated group compared with the control group. These results indicate that PP2A is a direct target protein of ATG.

### 3.6. ATG Improves Yeast Survival under Oxidative Stress

**Figure 6:**
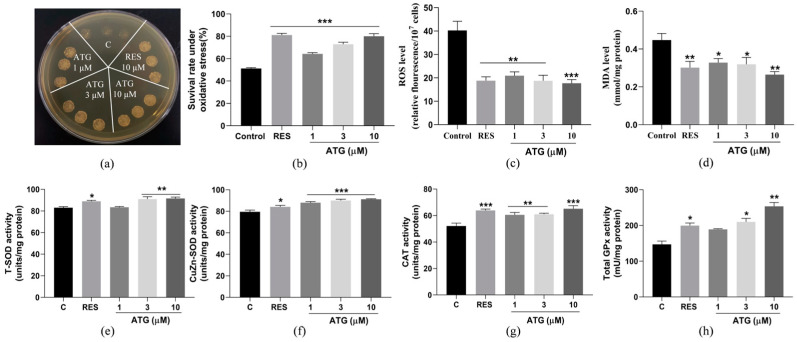
Effect of ATG on antioxidant capacity of yeast. (**a**) Survival status of BY4741 yeast cells under oxidative stress induced by 9.5 mM H_2_O_2_. (**b**) Quantification of yeast cell viability under 6.0 mM H_2_O_2_-induced oxidative stress. (**c**,**d**) ROS and MDA levels in yeast cells and (**e**–**h**) the effects of on T-SOD, CuZn-SOD, CAT, and GPx antioxidant enzyme activities in BY4741 yeast treated with 1, 3, and 10 µM ATG or RES (10 µM) for 24 h. *, **, and *** represent significant differences compared with the negative control group for *p* < 0.05, *p* < 0.01, and *p* < 0.001, respectively.

Oxidative stress can damage the normal physiological function of the body and is an important factor in promoting aging [14]. As shown in Figure 6a, the effect of ATG treatment on yeast colonies growth under 9.5 mM H_2_O_2_-induced oxidative stress. It can be clearly observed that under the influence of H_2_O_2_, the growth of yeast cells in the negative control group were inhibited, and the number of viable cells were greatly reduced. The growth status of yeast cells in the positive control group (10 µM RES) and ATG treatment groups were relatively normal, indicating that the treatment of 1, 3, and 10 µM ATG can improve the survival rate of yeast under oxidative stress. Approximately 200 yeast cells were dispersed on YPD agar plates containing 0 or 6.0 mM H_2_O_2_ to quantify yeast viability in oxidative stress condition and the survival rate of yeast was calculated by counting the number of colonies per group (Figure 6b). The survival rates of yeast in each group were as follows: negative control group: 51.24% ± 0.71%, positive control group (10 µM RES): 81.15% ± 1.44% (*p* < 0.001), 1 µM ATG group: 64.29% ± 1.09% (*p* < 0.001), 3 µM ATG group: 72.90% ± 1.80% (*p* < 0.001), and 10 µM ATG group: 80.05% ± 2.35% (*p* < 0.001). After ATG treatment, the survival rate of yeast was greatly increased. These results indicate that ATG had anti-aging activity by inhibiting oxidative stress.

In addition, ROS plays a key role in the physiological activities of organisms. However, when there is an imbalance between oxidation and antioxidation in the body, excessive accumulation of ROS will result in oxidative stress, which is harmful to the body [14]. MDA, one of the end products of polyunsaturated fatty acids after ROS degradation, is a substance that can mutate proteins to be toxic, which is a biomarker of oxidative stress [37]. Therefore, we reflected the anti-oxidative stress effect of ATG by testing its effect on ROS and MDA levels. The results of ROS levels were shown in Figure 6c, the ROS content decreased from 40.22 ± 3.95 to 18.79 ± 5.88 (*p* < 0.01) in the 10 µM RES group and ATG at doses of 1, 3, and 10 µM decreased ROS levels from 40.22 ± 3.95 to 20.92 ± 1.63 (*p* < 0.01), 18.78 ± 2.34 (*p* < 0.01), and 17.70 ± 1.56 (*p* < 0.001), respectively. Furthermore, as can be seen from Figure 6d, the MDA levels of yeast in the ATG-treated groups were also reduced relative to the negative control group. The MDA level in the positive group (10 µM RES) was decreased from 0.44 ± 0.01 to 0.30 ± 0.04 (*p* < 0.01) and it was decreased to 0.33 ± 0.26 (*p* < 0.05), 0.32 ± 0.04 (*p* < 0.05) and 0.26 ± 0.21 (*p* < 0.01) after treatment with 1, 3, and 10 µM ATG, respectively. These results suggest that ATG can resist oxidative stress by reducing the levels of ROS and MDA.

Antioxidant enzymes play an important role in fighting cellular oxidative stress, breaking down reactive oxygen species that are harmful to cells and reducing the damage caused by oxidative stress to the organism [12]. Therefore, we investigated the effects of different doses of ATG on T-SOD, CuZn-SOD, CAT, and GPx antioxidant enzymes in yeast cells. The activities of T-SOD (Figure 6e, *p* < 0.01 and *p* < 0.01), CuZn-SOD (Figure 6f, *p* < 0.001, *p* < 0.001, and *p* < 0.001), CAT (Figure 6g, *p* < 0.01, *p* < 0.01, and *p* < 0.001), and GPx (Figure 6h, *p* < 0.05 and *p* < 0.01) antioxidant enzymes were increased in yeast cells treated with ATG for 24 h. These findings show that ATG can resist oxidative stress by enhancing the activity of antioxidant enzymes, thereby achieving the effect of delaying aging.

### 3.7. Involvement of SOD1, SOD2, CAT, and GPx Genes in the Anti-Aging Effects of ATG

**Figure 7:**
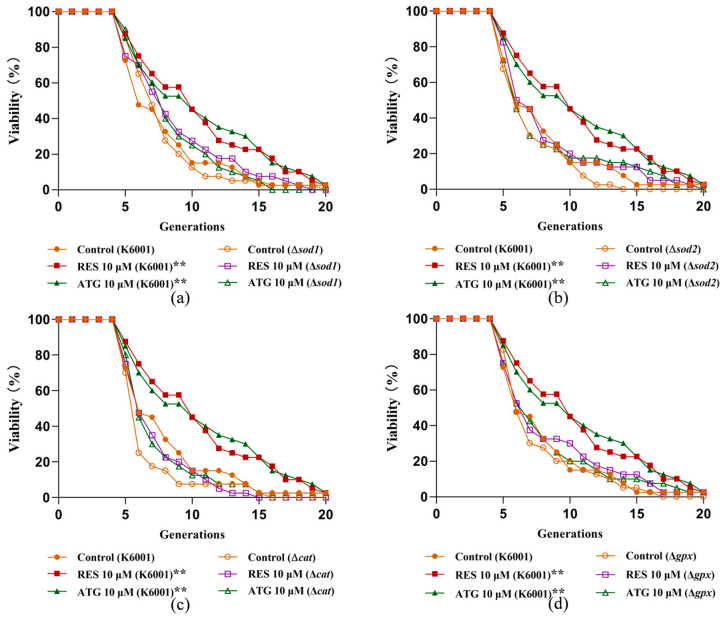
Effects of ATG on replicative lifespan of K6001 yeast mutants Δ*sod1* (**a**), Δ*sod2* (**b**), Δ*cat* (**c**), and Δ*gpx* (**d**). The average lifespan in K6001 were 7.03 ± 0.58 in negative control, 9.68 ± 0.75 in positive treatment group (*p* < 0.01), and 9.73 ± 0.80 in the group treated with 10 µM ATG (*p* < 0.01). (**a**) The average lifespan of Δ*sod1* mutant were 6.98 ± 0.48 in the negative group, 7.95 ± 0.61 in the positive group, and 7.70 ± 0.49 in the 10 µM ATG treatment group. (**b**) The average lifespan of Δ*sod2* mutant were 6.18 ± 0.39 in the negative group, 7.38 ± 0.64 in the positive group, and 6.78 ± 0.64 in the 10 µM ATG treatment group. (**c**) The average lifespan of Δ*cat* mutant were 5.75 ± 0.43 in the negative group, 6.35 ± 0.40 in the positive group, and 6.58 ± 0.53 in the 10 µM ATG treatment group. (**d**) The average lifespan of Δ*gpx* mutant were 6.78 ± 0.51 in the negative group, 7.58 ± 0.67 in the positive group, and 7.38 ± 0.66 in the 10 µM ATG treatment group. ** represent significant differences compared with the negative control group for *p* < 0.01.

*SOD1*, *SOD2*, *CAT*, and *GPx* are genes associated with antioxidants. *SOD1* and *SOD2* encode homologous proteins that convert ROS to H_2_O_2_ and thereby protect cells from oxidative damage, whereas *CAT* and *GPx* genes encode Cat and Gpx proteases that break down H_2_O_2_ into harmless H_2_O [12]. Therefore, we tested the effect of ATG on the replicative lifespan of the yeast mutant strains Δ*sod1*, Δ*sod2*, Δ*cat*, and Δ*gpx* with a K6001 background. As shown in Figure 7a,d, ATG could prolong the average lifespan of K6001 strain, but did not affect the replicative lifespan of Δ*sod1*, Δ*sod2*, Δ*cat*, and Δ*gpx* mutants with K6001 background. The above results indicated that the anti-aging effect of ATG is related to these antioxidant genes.

### 3.8. ATG Increased Telomerase Content and mRNA Expression of EST1, EST2 and EST3 in Yeast

**Figure 8:**
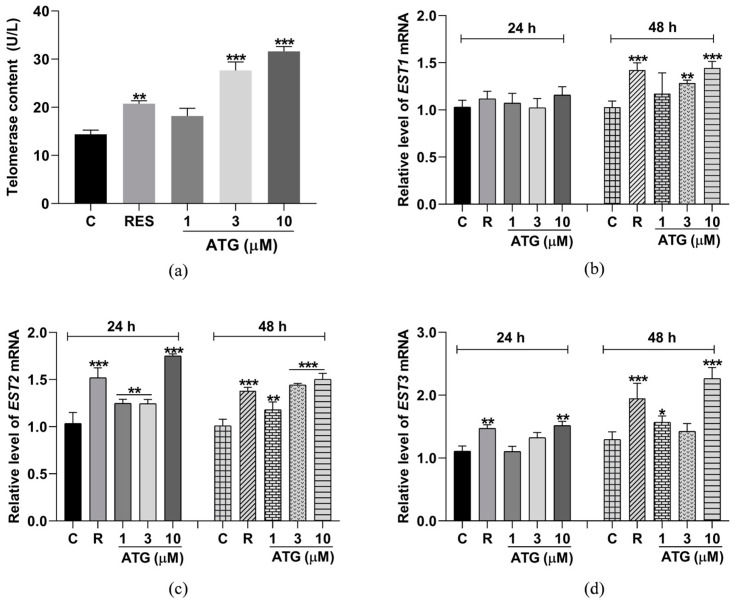
Effect of ATG on S288C yeast telomerase. (**a**) Effect of ATG treatment on yeast telomerase content. (**b**–**d**) Effect of ATG treatment on the expression of telomerase-related genes EST1, EST2, and EST3 for 24 h or 48 h. *, **, and *** represent significant differences compared with the negative control group for *p* < 0.05, *p* < 0.01, and *p* < 0.001, respectively.

Telomerase activity is closely related to cellular senescence and age-related diseases [15]. Therefore, we assessed whether ATG was associated with yeast telomerase activity. The results showed that telomerase content was increased in yeast cells after 48 h treatment with ATG at concentrations of 3 µM and 10 µM (Figure 8a, *p* < 0.001, and *p* < 0.001). To explore whether the ATG-mediated telomerase activity is related to its effect on the expression of telomerase-related genes, we measured the transcript abundance of EST1, EST2, and EST3 in yeast after 24 or 48 h of treatment with different doses of ATG using qRT-PCR. The results showed that the mRNA levels of EST1 were significantly increased in cells treated with ATG for 48 h, but there was no significant difference in its content in yeast treated for 24 h (Figure 8b, *p* < 0.01, and *p* < 0.001). Meanwhile, the transcript levels of EST2 (Figure 8c, *p* < 0.01, *p* < 0.01, *p* < 0.01, *p* < 0.001, *p* < 0.01, *p* < 0.001, and *p* < 0.001), and EST3 (Figure 8d, *p* < 0.01, *p* < 0.05, and *p* < 0.001) were increased in cells treated with ATG for 24 h or 48 h, respectively. These results suggested that ATG may enhance telomerase content and activity in yeast by mediating the transcription of EST1, EST2, and EST3 genes, thereby extending lifespan.

## 4. Discussion

Natural plants stand as the important sources for drug development, yielding a plenty of small molecules with anti-aging activity including RES, Rapa, and quercetin [3,37]. *Fructus arctii* possess particularly great medicinal value for its rich content of lignans, which shows various biological activities such as lowering blood glucose, anti-cancer, anti-oxidation, and anti-inflammation [23,38]. In addition, the total lignans in *Fructus arctii* are reported to prolong the lifespan of *C. elegans*. However, the main anti-aging molecule and the underlying mechanisms still remain unclear. Therefore, it is necessary to investigate the target natural molecule in *Fructus arctii* responsible for the main anti-aging effect and to study its mechanism of action.

Senescent cells are an important factor in aging and the development of a variety of age-related diseases. Therefore, the development of senotherapeutics that can specifically kill senescent cells or slow down cells aging rate is very effective for delaying aging [4]. In this study, guided by the replicative lifespan of K6001, we screened for a compound, ATG, capable of extending yeast lifespan and slowing Eto-induced mammalian cells senescence (Figure 1). It exhibits the best anti-aging activity in yeast at a dose of 10 µM and in mammalian cells at a dose of 30 µM. On this basis, we further evaluated the anti-aging mechanism of ATG using MTT and Western blot (Figure 2) and found that ATG was a senomorphic that slowed down the degree of cellular senescence by mediating p21 and p53 pathways. Researches show that ATG is a key compound contributing to the pharmacological effects of *Fructus arctii* in the prevention of various age-related diseases, including its ability to alleviate AD symptoms by reducing β-amyloid (Aβ) formation and senile plaques [25], promote glioma cell apoptosis by increasing autophagy, and regulate the immune system to slow the progression of multiple sclerosis [38]. The anti-aging effect of ATG is considered to relate to these pharmacological effects, which provides us with ideas for further study on the mechanism of ATG in prolonging lifespan.

Autophagy is known to maintain cell homeostasis and is closely intertwined with the aging process. The accumulation of abnormal proteins in several age-related neurological diseases is linked to impaired autophagy [25]. In addition, the level of autophagy decreases as cells age, and the activation of autophagy has been reported to prolong the lifespan of *Drosophila melanogaster* [6,25]. Therefore, it is worth exploring the relationship between autophagy and the anti-aging effect of ATG on yeast lifespan. ATG treatment in the dose range of 1–10 µM significantly increased the free GFP flux in YOM38-*GFP-ATG8* yeast and had no effect on the lifespan of yeast mutants Δ*atg2* and Δ*atg32* with deletion of autophagy-related genes in Figure 3, confirmed that autophagy plays a key role in the anti-aging activity of ATG. At the same time, previous studies have shown that ATG targets PP2A to alleviate diabetic nephropathy when the methylation level of PP2A is closely related to brain aging in stone cystitis monkeys [29,39]. Since ATG at a dose of 10 µM had the best autophagic activity, this concentration was chosen to study the relationship between PP2A and its anti-aging effect (Figure 4 and Figure 5). It was found that PP2A was a direct target protein of ATG, and ATG could induce autophagy by activating PP2A. Both slowing down cellular senescence and enhancing autophagy are important approaches for the treatment of age-related diseases, and ATG can not only slow down senescence but also promote autophagy. A class of small molecules like this, along with fenofibrate, minoxidil, tomatine, and astemizole, represent a new generation of compounds [34].

Oxidative stress caused by excessive accumulation of ROS is often a major cause of various age-related diseases such as neurodegenerative diseases, cardiovascular diseases, and aging [14]. The deficiency of antioxidants or the excess of oxide substances are the main factors leading to ROS accumulation [5]. Therefore, exploring small molecules with antioxidant effects emerges as an effective strategy to delay aging [7,23]. Lignans containing phenolic hydroxyl groups generally exhibit good antioxidant activity [23]. Our results showed that ATG in the 1–10 µM range significantly prolonged survival rate of yeast under oxidative stress by targeting genes related to antioxidant enzymes, increasing antioxidant enzyme activity, and reducing ROS and MDA levels (Figure 6 and Figure 7). It is worth mentioning that the most abundant lignan in *Fructus arctii* is arctiin, a glycoside of ATG, which is about 10–20 times more abundant than ATG. It can be converted into ATG under the action of human intestinal flora, so as to have antioxidant effect in vivo [26]. In the subsequent work, we will investigate the relationship between the antioxidant effects of arctiin and ATG, as well as the association between arctiin and longevity.

At the same time, telomere loss is one of the hallmarks of aging. Many age-related chronic diseases such as AD, coronary heart disease and type 2 diabetes are associated with telomere shortening [15]. Telomerase not only protects telomeres from depletion, but also promotes angiogenesis, improves metabolism, protects mitochondria, and regulates gene expression [15]. Thus, drug development based on telomerase and telomeres is beneficial to combat aging as well as chronic diseases. At present, a number of active molecules have been isolated from traditional Chinese medicine, including astragaloside, garlicin, and cistanche tubulose acteoside, which are capable of resisting aging by increasing telomerase activity or protecting telomeres [16]. In this study, we investigated the association between ATG and telomerase and found that ATG in the range of 1–10 µM increased telomerase content and the expression of related genes *EST1*, *EST2*, and *EST3* (Figure 8), indicating that ATG could enhance telomerase activity in yeast.

## 5. Conclusions

**Figure 9:**
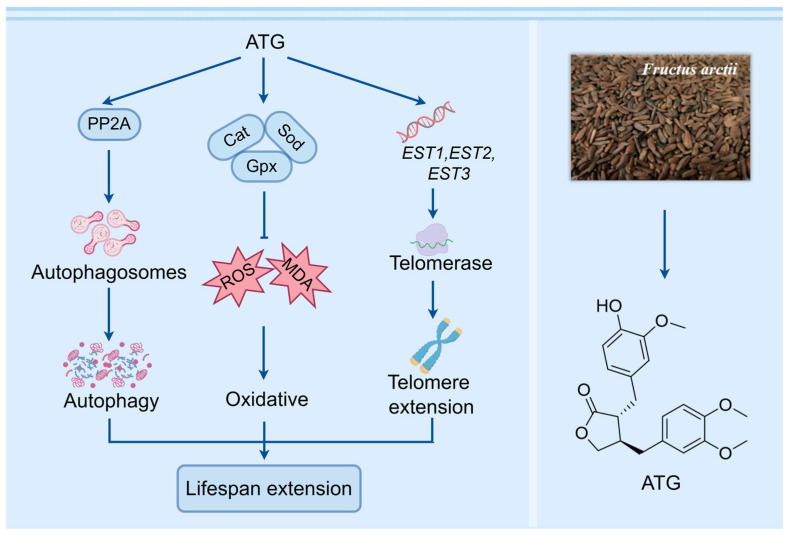
The proposed mechanisms of antiaging by ATG. ATG prolongs lifespan by induction of autophagy, anti-oxidative stress, and enhancement of telomerase activity in yeast.

In summary, ATG from *Fructus arctii* exerts anti-aging effects through multiple signaling pathways, including induction of autophagy, anti-oxidative stress, and enhancement of telomerase activity (Figure 9). This study provides an important basis for the deeper study of the anti-aging mechanism of ATG. In the near future, we will further explore the anti-aging effect of ATG in animal models and focus on the relationship of chemical structure and activity to conduct deep research work.

## Data Availability

All figures and data used to support this study are included within this article.

## Supplementary Materials

The following supporting information can be downloaded at https://www.mdpi.com/article/10.3390/antiox13060684/s1. The measurement of SOD, CAT, and GPx enzymatic activity are shown in Supplementary Methods. The specific ratios of amino acids and nucleotides in SC medium are shown in Supplementary Table S1; the genotypes of yeast strains and mutants are described in Supplementary Table S2. The ^1^H NMR spectrum of ATG is shown in Supplementary Figure S1; the replicative lifespan of K6001 yeast at concentrations of 0.3, 1, 3, 10, 30, and 100 µM of ATG are shown in Supplementary Figure S2; the full unedited gel for Western blot analysis of p16, p21, and p53 in Figure 2 are shown in Supplementary Figure S3; the full unedited gel for Western blot analysis of free GFP and β-actin of yeast in Figure 3 are shown in Supplementary Figure S4; the full unedited gel for Western blot analysis of PP2A of DARTS and CETSA in Figure 5 are shown in Supplementary Figure S5.

## Abbreviations

AD	Alzheimer’s disease
ATG	Arctigenin
CAT	Catalase
CETSA	Cellular thermal shift assay
DARTS	Drug affinity responsive target stability
EdU	5-Ethynyl-2′-deoxyuridine
Eto	Etoposide
GPx	Glutathione peroxidase
MDA	Malondialdehyde
MTT	3-(4,5-Dimethylthiazol-2-yl)-2,5-diphenyltetrazolium bromide
PBS	Phosphate buffer
PP2A	Protein phosphatase 2A
Rapa	Rapamycin
ROS	Reactive oxygen species
RES	Resveratrol
SASPs	Senescence-associated secretory phenotypes
SA-β-Gal	Senescence-associated β-galactosidase
SOD	Superoxide dismutase
SC	Synthetic complete glucose
SD	Synthetic defined
YPD	Yeast peptone dextrose
