# Supplementary material for: Arctigenin from Fructus arctii Exhibits Antiaging Effects via Autophagy Induction, Antioxidative Stress, and Increase in Telomerase Activity in Yeast
Source: Antioxidants (Basel). 2024 Jun 2;13(6):684. doi: 10.3390/antiox13060684 (PMC11200627; doi:10.3390/antiox13060684)
Supplement: Supplementary file 1 [file antioxidants-13-00684-s001.zip › antioxidants-3036391-supplementary.pdf]

Supplementary Information

Arctigenin from *Fructus arctii* Exhibits Antiaging Effects via Autophagy Induction, Antioxidative Stress, and Increase in Telomerase Activity in Yeast

Siqi Chen [1], Yajing Li [2], Enchan Wu [2], Qing Li [1,*], Lan Xiang [2,*] and Jianhua Qi [2,*]

[1] College of Chemistry and Materials Science, Sichuan Normal University, Chengdu 610068, China; 20221201040@stu.sicnu.edu.cn

[2] College of Pharmaceutical Sciences, Zhejiang University, Yu Hang Tang Road 866, Hangzhou 310058, China; 12019045@zju.edu.cn (Y.L.); 22019019@zju.edu.cn (E.W.)

* Correspondence: qingli2021@sicnu.edu.cn (Q.L.); lxiang@zju.edu.cn (L.X.); qijianhua@zju.edu.cn (J.Q.)

1. Supplementary Materials

Analyze pure reagents (methanol and ethyl acetate were bought from Sinopharm Chemical Reagent Co., Ltd., Shanghai, China; n-hexane was from Huzhou Shuanglin Chemical Technology Co., LTD., Zhejiang, China). Silica gel (200-300 mesh, Yantai Research Institute of Chemical Industry, Yantai, China) and Cosmosil 5C_{18}-MS-Ⅱ packed column (Nacalai Tesque, Japan) were used for the isolation and purification of natural products. TLC analysis was performed using thin layer chromatography silica gel precast plates (Beijing Jianqiang Weiye Technology Co., Ltd., Beijing, China) and RP-18 F254s 25 glass plates (Yantai Jiangyou Silicone Gel Development Co., Ltd., Yantai, China). $CDCl_3$ (Qiaoyi Biotechnology Shanghai Co., Ltd., Shanghai, China) was used as a ^{1}H NMR solvent. The ^{1}H NMR spectra were obtained using a Bruker AV Ⅲ-500 spectrometer (Bruker, Karlsruhe, Germany). The compounds and reagents listed below were used in biological experiments: Hoechst 33342 (Mackiln, Shanghai, China), RES (J&K Scientific Ltd., Beijing, China), rapamycin (Solarbio, Beijing, China), LB-100 (Shanghai Biyuntian Biotechnology Co., Ltd., Shanghai, China). Ethanol and DMSO were used as a solvent to dissolve the compounds or as a negative control for yeast-related and PC12 cells experiments, respectively.

2. Supplementary Methods

Measurement of SOD, CAT and GPx Enzyme Activities

According to the instructions of the SOD assay kit (Nanjing Jiancheng Bioengineering Institute, Nanjing, China), 25 μg protein in each group was first mixed with reagent VII and vortexed for 1 min to inactivate the Mn-SOD enzyme in the samples. The supernatant was obtained for the detection of the CuZn-SOD enzyme activity after centrifugation (3000× g, 15 min). The reagent I and blank control samples, and the samples treated by reagent VII were added to the 96-well plate according to the dosage on the instructions. Then, reagents II, III, and IV were added into each well. Then, the plate was incubated at 37 °C for 40 min after mixing well. Finally, the A_{550} absorbance value of the samples was measured after reacting with 200 μL chromogenic working fluid at room temperature for 10 min. The activity of SOD enzyme = ([control group OD value - determination group OD value]/control group OD value)/50% × (total volume of reaction solution/sample volume)/protein concentration of sample.

The method of CAT enzyme activity assay was following the manufacturer's instructions for the CAT assay kit (Beyotime Biotech, Shanghai, China). Briefly, gradient concentrations of hydrogen peroxide solution were first prepared. Afterward, chromogenic working fluid was added into the 96-well plate to mix with hydrogen peroxide solution and reacted at 25 °C for 15 min. The standard curve of the hydrogen peroxide concentration was determined after measuring the absorption value at 520 nm. Simultaneously, catalase buffer and 250 mM of hydrogen peroxide were added to each well along with 8 μL protein (1.25 μg/μL). After reacting at 25 °C for 1–5 min, 450 μL enzyme reaction termination solution was added to terminate the reaction. Then, 10 μL of the mixture was taken to react with chromogenic working fluid at 25 °C for 15 min, and the absorption value of A_{520} was measured. The sample catalase activity = [consumption of micromole of hydrogen peroxide] × [dilution ratio]/([reaction minutes] × [sample volume] × [protein concentration]), and [consumed micromole of hydrogen peroxide] = [micromole of residual hydrogen peroxide in blank control] − [micromole of residual hydrogen peroxide of sample].

For the GPx enzyme activity measurement, all the procedures followed the instructions of the GPx assay kit (Beyotime Biotech, Shanghai, China). Almost 5 μg protein of each sample was taken. The general process is that the GPx detection buffer, samples, GPx detection working solution, and peroxide reagent were added to a 96-well plate in turn. The absorbance value of the A_{340} was measured every 4 min, six times, after mixing well. The activity of GPx in the detection system = [(ΔA_{340} (sample) − ΔA_{340} (blank))/min]/ (0.00622 μM^{-1} cm^{-1} × 0.276 cm). Total GPx activity in the sample = GPx activity in the detection system × dilution ratio/sample protein concentration.

3. Supplementary Tables

Table S1. The specific ratios of amino acids and nucleotides in SC medium.

Component	mg/L
Adenine	100
Uracil	100
L-Arginine	100
L-Cysteine	100
L-Lysine	100
L-Threonine	100
L-Tryptophan	100
L-Leucine	100
L-Glutamic acid	100
L-Aspartic acid	50
L-Isoleucine	50
L-Phenylalanine	50
L-Serine	50
L-Tyrosine	50
L-Valine	50
L-Methionine	50
L-Histidine	50

Table S2. Yeast strains used in the present study.

Strains	Genotype	Source
K6001	*MATa*, *ade2-1*, *trp1-1*, *can1-100*, *leu2-3,112*, *his3-11,15*, *GAL*, *psi+*, *ho*::HO::*CDC6* (at HO), *cdc6*::*hisG*, *ura3*::*URA3 GAL-ubiR-CDC6* (at URA3)	Gifted by Professor Michael Breitenbach
Δ*sod1*, Δ*sod2*, Δ*cat*, Δ*gp*x, Δ*atg2*, Δ*atg32* of K6001	Replace the *SOD1* gene, *SOD2* gene, *CAT* gene, *GPx* gene, *ATG2* gene, and *ATG32* gene in K6001 with kanamycin gene, respectively	
BY4741	*MATa*, *his3Δ1*, *leu2Δ0*, *met15Δ0*, *ura3Δ0*	Constructed by Professor Akira Matsuura
YOM38 containing pR316-*GFP-ATG8* plasmid	Prototrophic derivative of BY4742 (*MATα*, *his3Δ1*, *leu2Δ0*, *lys2Δ0*) containing plasmid pRS316-*GFP-ATG8*	
S288C	*MATα*, *SUC2*, *gal2*, *mal2*, *mel*, *flo1*, *flo8-1*, *hap1*, *ho*, *bio1*, *bio6*	Purchased from Hangzhou Baosai Biotechnology Co., LTD

4. Supplementary Figures

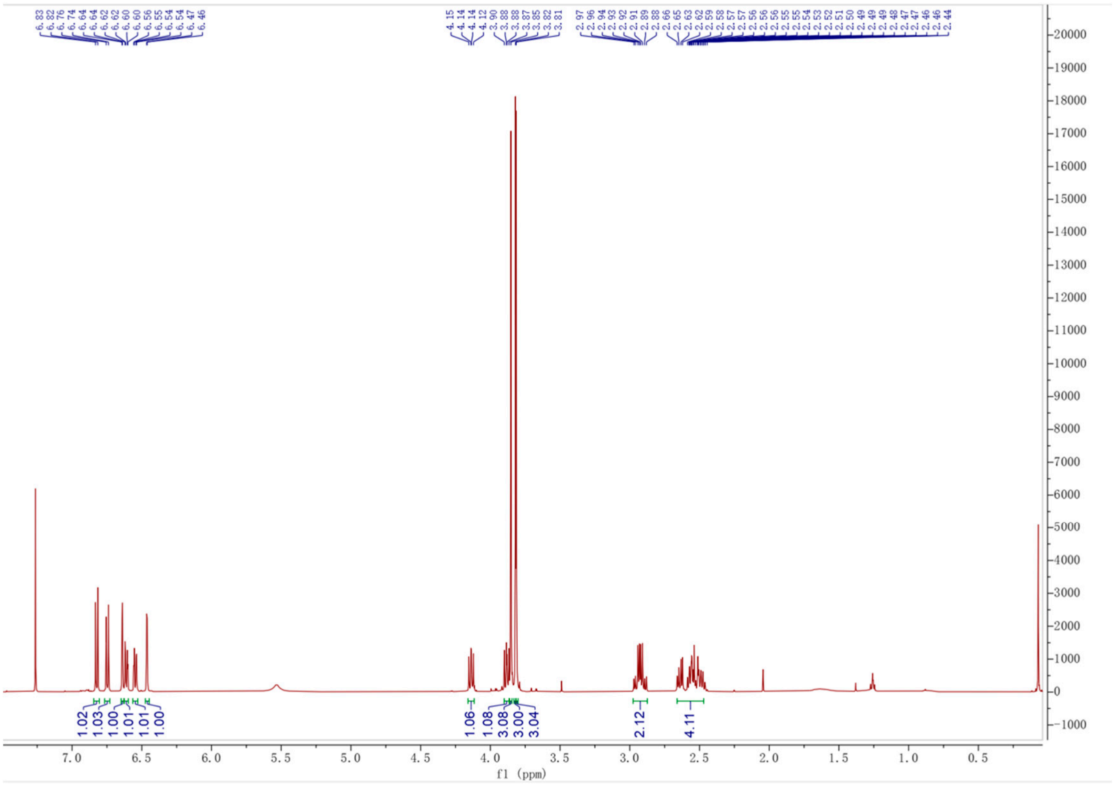

Supplementary Figure S1. The ^{1}H NMR spectrum of ATG (500 MHz, $CDCl_3$)

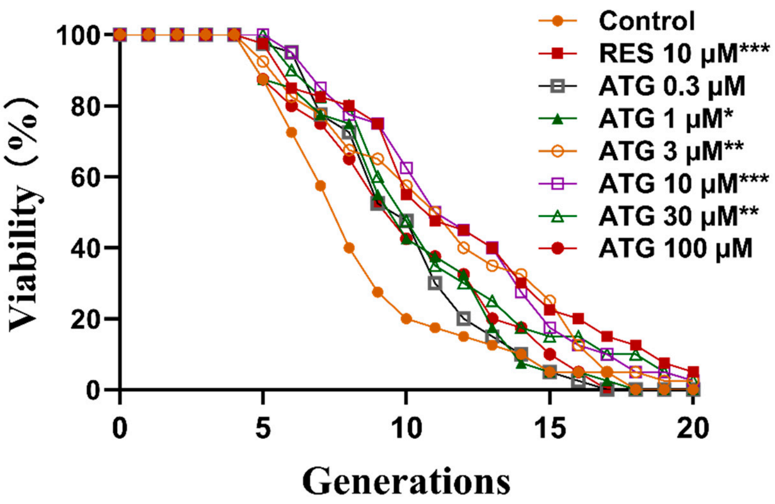

Supplementary Figure S2. Effect of ATG on the replicative lifespan of K6001 yeasts. The average lifetime of each group was as follows: control (7.75 ± 0.54), RES at a dose of 10 μM (11.20 ± 0.71), ATG at a dose of 0.3 μM (8.64 ± 0.55), ATG at a dose of 1 μM (9.30 ± 0.53), ATG at a dose of 3 μM (10.53 ± 0.69), ATG at a dose of 10 μM (11.10 ± 0.61), ATG at a dose of 30 μM (10.25 ± 0.63), ATG at a dose of 100 μM (9.00 ± 0.56). *, ** and *** represent significant differences compared to the control group at $p < 0.05$, $p < 0.01$, and $p < 0.001$, respectively.

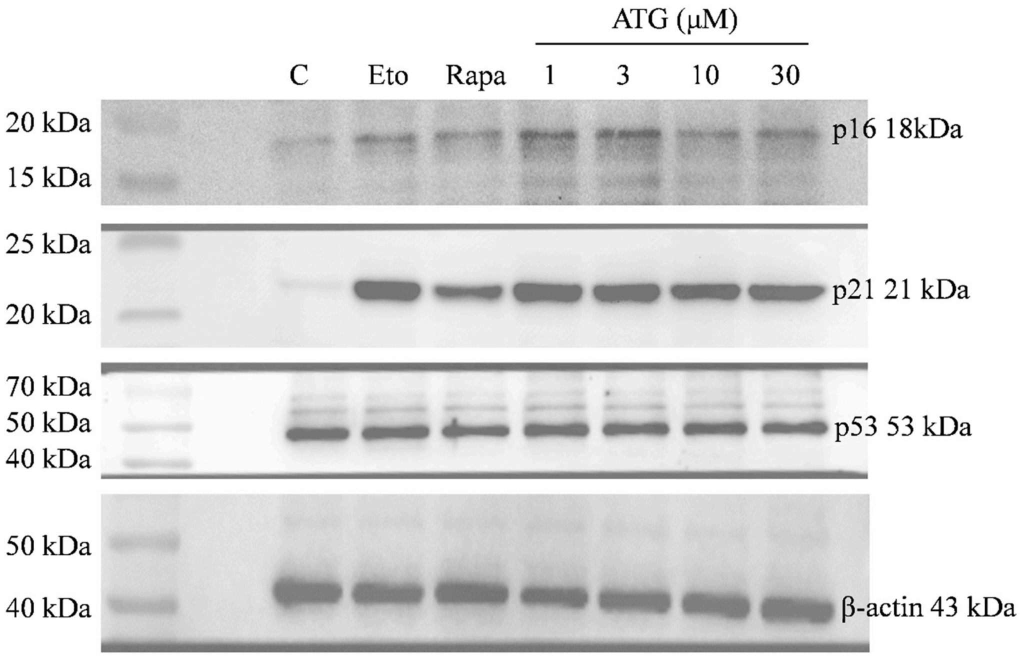

Supplementary Figure S3. Original data of Western blot analysis of p16, p21, p53, and β-actin in Figure 2c.

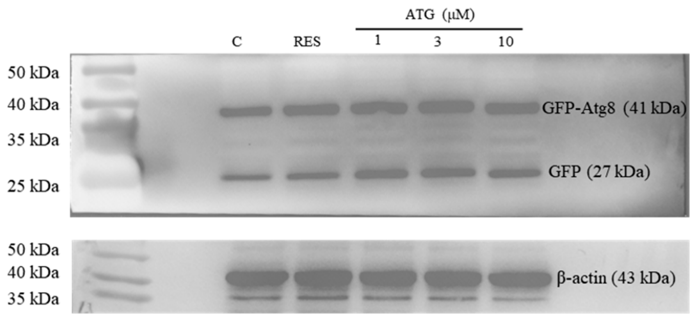

(a)

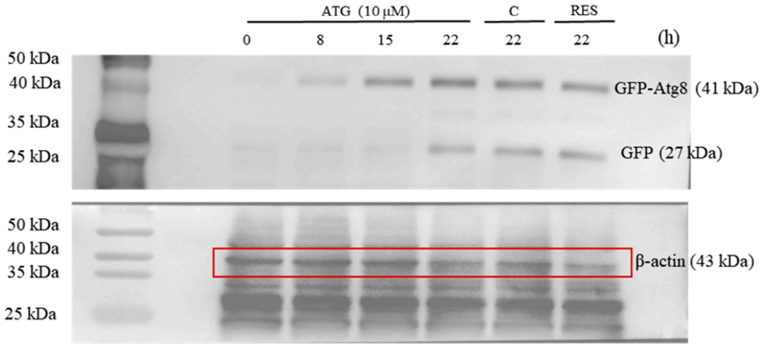

(b)

Supplementary Figure S4. Original data of Western blot analysis of free GFP and β-actin in Figures 3c and 3e. (**a**) Original data in Figure 3c present the effect of ATG on autophagy. (**b**) Original data in Figure 3e show the time-course of autophagy upon ATG treatment.

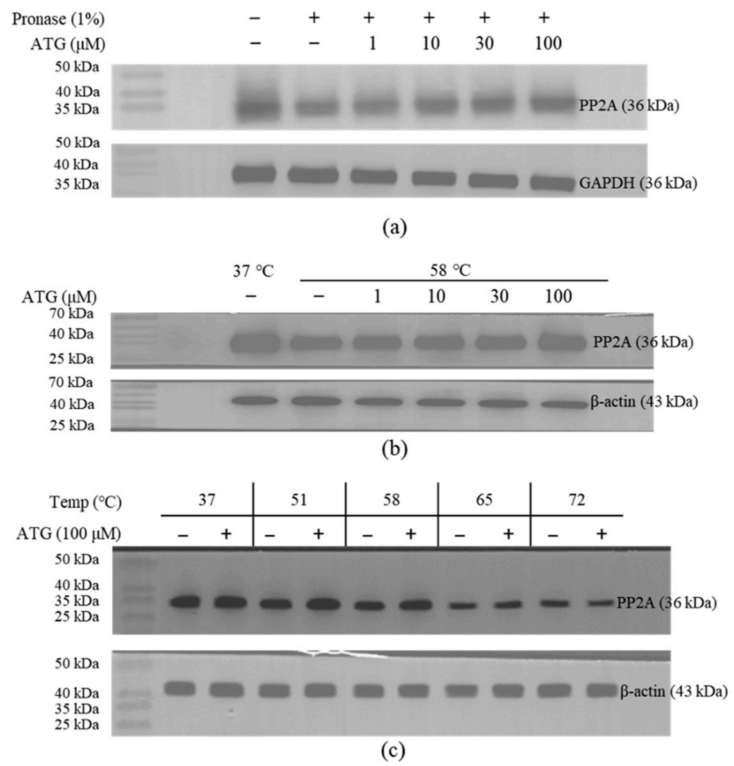

Supplementary Figure S5. Original data of Western blot analysis of PP2A, GAPDH, and β-actin in Figure 5. (a) Original data in Figure 5a present the changes in the protein PP2A in NIH/3T3 cells after administering of various concentrations of ATG and digesting with pronase E. (b) Original data in Figure 5c present the changes in the protein PP2A in NIH/3T3 cells after administering of various concentrations of ATG and heating the samples at 58 °C. (c) Original data in Figure 5e present the changes in the protein PP2A in NIH/3T3 cells after treating with ATG at a dose of 100 μM and heating the samples at different temperatures.
